# Specific Conditions for Resveratrol Neuroprotection against Ethanol-Induced Toxicity

**DOI:** 10.1155/2012/973134

**Published:** 2012-06-17

**Authors:** Brigitte Gonthier, Nathalie Allibe, Cécile Cottet-Rousselle, Frédéric Lamarche, Laurence Nuiry, Luc Barret

**Affiliations:** ^1^INSERM, U1055 (LBFA), Université Joseph Fourier, F-38041 Grenoble, France; ^2^CHRU Grenoble, Hôpital Michallon, Service de Médecine Légale et Toxicologie, F-38043 Grenoble, France; ^3^Laboratoire de Médecine Légale et Toxicologie, Université Joseph Fourier, F-38041 Grenoble, France

## Abstract

*Aims*. 3,5,4′-Trihydroxy-*trans*-stilbene, a natural polyphenolic compound present in wine and grapes and better known as resveratrol, has free radical scavenging properties and is a potent protector against oxidative stress induced by alcohol metabolism. Today, the mechanism by which ethanol exerts its toxicity is still not well understood, but it is generally considered that free radical generation plays an important role in the appearance of structural and functional alterations in cells. The aim of this study was to evaluate the protective action of resveratrol against ethanol-induced brain cell injury. *Methods*. Primary cultures of rat astrocytes were exposed to ethanol, with or without a pretreatment with resveratrol. We examined the dose-dependent effects of this resveratrol pretreatment on cytotoxicity and genotoxicity induced by ethanol. Cytotoxicity was assessed using the MTT reduction test. Genotoxicity was evidenced using single cell gel electrophoresis. In addition, DNA staining with fluorescent dyes allowed visualization of nuclear damage using confocal microscopy. *Results*. Cell pretreatment with low concentrations of *trans*-resveratrol (0.1–10 **μ**M) slowed down cell death and DNA damage induced by ethanol exposure, while higher concentrations (50–100 **μ**M) enhanced these same effects. No protection by *cis*-resveratrol was observed. *Conclusion*. Protection offered by *trans*-resveratrol against ethanol-induced neurotoxicity was only effective for low concentrations of this polyphenol.

## 1. Introduction

The brain is particularly susceptible to oxidative stress due to its high rate of oxygen consumption, its high proportion of polyunsaturated fatty acids and its low levels of antioxidant defense enzymes [1]. Consequently, the brain is a first-class target in situations in which free radicals are implicated such as ageing, neurodegenerative diseases, and xenobiotic metabolism. This organ is thus a major target for ethanol, and its consumption has long been associated with severe damage. The adverse effect of ethanol on different functions of the central nervous system has been well documented. Numerous experimental studies and necropsy examinations have shown a wide range of structural and functional alterations in neurons as well as in astrocytes. Astrocytes were chosen as a model for cerebral intoxication because their deleterious implication has considerable potential consequences. First, they represent the most abundant cell type in the central nervous system. Besides providing nutrients and neurotrophic factors to neurons, these cells are immune-active and actively participate in host defense mechanisms [2, 3]. In addition, specific enzyme systems allow astrocytes to metabolize xenobiotics, free radicals, and metals, thus protecting the brain from the toxicity of these agents [4, 5].

The advantage of primary cultures lies in their closer resemblance to cells found *in vivo*. The drawbacks of this choice are their limited survival time and the continual need to prepare new cells in culture. This can explain why very few reports are found in the literature on this subject.

There is strong evidence showing that chronic or excessive ethanol consumption enhances oxidative damage to brain. This is due to the ability of ethanol to increase oxidative stress, leading to enhanced production of oxidative species including reactive oxygen species (ROS) and in the formation of lipid peroxidation products [6, 7].

Using the spin-trapping technique, we previously detected hydroxyethyl-free radical formation following ethanol exposure of various biological systems such as rat liver or brain microsomes [8] and primary culture of rat astrocytes or C6 glioma cell line [9].

ROS are produced during normal cell metabolism as well as after exposure to various xenobiotics including alcohol. Although GSH and various antioxidant enzymes (SOD, GPX, catalase, etc.) prevent cells from attack by these ROS, massive or chronic exposure to a toxic substance may induce an oxidant/antioxidant imbalance, leading to cellular oxidative damage. The literature has reported that antioxidant micronutrients such as vitamin C, vitamin E, and *β*-carotene in fruits and vegetables contribute in part to protecting cells against the damaging effects of ROS.

3,5,4′-Trihydroxy-*trans*-stilbene, so called resveratrol, is a naturally occurring stilbene found in the skin of red grapes and certain medicinal plants, where it is believed to provide protection against various infections and stresses [10]. In addition, this red wine constituent may be implicated in the so-called French paradox, a phenomenon relating the low incidence of coronary heart disease in southern France despite a high dietary intake of saturated fats [11]. Numerous beneficial health effects have been reported such as anticancer, antiviral, antiinflammatory, antiageing and neuroprotective effects [12–15]. Indeed, neuroprotective effects have been related in cerebral ischemia models as well as in neurodegenerative diseases such as Alzheimer's disease, Huntington's disease, and Parkinson's disease [16, 17]. Moreover, most of the protective biological actions associated with resveratrol have been related to its intrinsic radical scavenger properties [18–21]. De Almeida et al. [22] demonstrated the protective effect of resveratrol against acute H_2_O_2_-induced oxidative stress in astrocyte cultures.

Since a free radical pathway is implicated in the cellular damage observed after ethanol exposure, we studied resveratrol pretreatment of astrocytes because this antioxidant might be able to promote the survival of brain cells exposed to ethanol stress.

The aim of this study was to evaluate the ability of resveratrol to protect primary culture of rat astrocytes against ethanol-induced cellular damage in a single model of long-term exposed cells through *in vitro* experiments. 

## 2. Materials and Methods

### 2.1. Culture of Primary Astrocytes

Pregnant Sprague-Dawley rats were obtained from Janvier (L'Arbresle, France). Animal care and use and all procedures involving animals were carried out in accordance with French national regulations.

The primary astrocyte cultures were prepared aseptically from cerebral hemispheres of 1- or 2-day-old pups, according to previously described methods [23] with a few modifications [9]. The dissociated cells were plated either in poly-L-lysine-coated 35-mm-diameter Petri dishes or in 75 cm^2^ plastic flasks at a density of 500 000 viable cells/mL in the usual D-MEM medium containing 10% FCS, 100 U/mL penicillin, 0.1 mg/mL streptomycin, and 2.5 *μ*g/mL amphotericin B. The cultures were maintained at 37°C in a 5% CO_2_-humidified atmosphere. The medium was changed every 3 days. After 12 days *in vitro* (DIV), the monolayers were confluent and composed of 95% astrocytes, as demonstrated by positive immunostaining with antiserum to *α*-GFAP, an astrocyte marker [24].

For confocal microscopy experiments, ten days after plating, cells in flasks were trypsinized and reseeded in the same medium at a density of 250 000 viable cells/mL, onto poly-L-lysine-coated 40-mm diameter glass coverslips. Cells were used 48 hours after seeding when culture reached 80–90% confluence.

All experiments were conducted in culture medium and in an air/CO_2_ incubator between 12 and 15 days *in vitro*.

### 2.2. Formation and Identification of *Cis*- and *Trans*-Isomers of Resveratrol

*Trans*-resveratrol was purchased from Sigma-Aldrich Chemical (Saint Louis, MO, USA).

Two 100-mM solutions of* trans*-resveratrol were prepared in H_2_O/DMSO (50/50). One aliquot was kept in the dark and the other placed to laboratory light, in order to induce the *trans-cis* isomerization. Then samples were derivatized for 30 min at 65°C using BSTFA (N,N-bis(trimethylsilyl)trifluoroacetamide). Finally, samples were analyzed by gas chromatography coupled to mass spectrometry (GC/MS). 

### 2.3. Conditions for Exposure to Resveratrol and/or Ethanol

*Trans*-resveratrol was dissolved in H_2_O/DMSO (50/50) so as to obtain a 100-mM stock solution which was filtered, then aliquoted and stored at −20°C until use. Just before the beginning of experiments, the *trans-cis* isomerization was realized by exposing an aliquot to laboratory light. Different intermediate dilutions of these two isomers were then prepared in the same mixture in order to preserve the same final H_2_O/DMSO/culture medium ratio for each resveratrol concentration tested. 

Cells were preincubated for 1 hour in the culture medium containing various concentrations of *cis-* or *trans-*resveratrol ranging from 0.1 to 100 *μ*M. Then, medium was replaced by a new one containing the same resveratrol concentration and 20 mM ethanol for three days. Controls with resveratrol alone or ethanol alone were also realized.

To avoid alcohol evaporation, we used a previously described compensating system, which provided a constant concentration of alcohol in the culture medium for 3 days [25].

### 2.4. Gas Chromatography/Mass Spectrometry Analysis

The analyses were performed on an Agilent Technologies 6890N Network GC System combined with an Agilent Technologies 5975 network mass selective detector and an Agilent Technologies 7683 series injector. The entire process, including data collection, was controlled by the Agilent Technologies Chem Station Version Rev.D.02.00.275.

We injected 2 *μ*L of each sample on to a DB-5 ms column (30 × 0.25 mm i.d.; 0.25-*μ*m film thickness) using pulsed splitless injection with an injector temperature of 250°C. Temperature conditions were as follows: initial temperature of 100°C for 1 min, increased to 300°C at 20°C/min, and held for 11 min. The flow of the carrier gas (helium) was maintained at 1 mL/min in constant flow mode. The gas chromatograph interface temperature was held at 315°C. Electron impact ionization was performed at 70 eV, with an ion source temperature of 230°C and mass spectra collected from 40 to 600 *m/z*. 

### 2.5. Determination of Cell Viability

After each treatment, cell viability was determined using the MTT reduction test [26]. Growth medium was replaced by D-MEM containing 0.5 mg/mL 3-(4,5-dimethylthiazol-2-yl)-2,5-diphenyltetrazolium bromide (MTT). After 2 h of incubation, cell medium was removed and replaced by 1 mL of dimethylsulfoxide to solubilise the precipitated formazan.

Cell viability was quantified spectrophotometrically at 540 nm, and 100% viability was assigned to the absorbance of control. At least five dishes were tested for each intoxication condition. The MTT assay, measuring the mitochondrial dehydrogenase activity, reflects the metabolic activity of the cells and is a helpful indicator of cell viability.

### 2.6. Evaluation of Nuclear Damage

#### 2.6.1. Comet Assay

DNA damage was evaluated using single cell gel electrophoresis, also called the comet assay [27]. This technique was realized using an electrophoresis power supply (Consort EV265) and a submarine horizontal electrophoresis system (Model HU25). This assay was performed either immediately after the stress to evidence damage generated by ethanol or after a 1- or 3-h recovery period with the aim of studying repair mechanisms. Therefore, the effects on DNA observed in these conditions reflect the initial DNA damage in terms of strand breaks and oxidatively damaged bases generating alkali-labile sites in DNA.

The procedure used was a modification of the protocol described by Singh et al. [28]. Frosted microscope slides were first covered with 150 *μ*L of 1% normal agarose in Ca^2+^- and Mg^2+^-free phosphate-buffered saline (PBS) and immediately covered with a 22 × 50 mm coverslip and kept at room temperature to allow the agarose to solidify. The coverslip was then gently slid off. Approximately 20 000 cells were suspended in 80 *μ*L of 0.8% low-melting-point agarose in PBS kept at 37°C and transferred onto the first agarose layer. After having been covered with a coverslip, the slides were left on ice for 5 min. Then the coverslips were removed and the slides were placed in freshly prepared lysing solution at 4°C for 1 h in the dark (2.5 M NaCl, 100 mM Na_2_EDTA, 10 mM Tris, 1% sodium sarcosinate, pH set to 10 with NaOH, supplemented with 10% DMSO and 1% Triton X-100 just before use). After lysis, the slides were gently transferred to a horizontal gel electrophoresis tank filled with freshly prepared electrophoresis solution (0.3 M NaOH, 1 mM Na_2_EDTA) at room temperature in the dark. The DNA was allowed to unwind for 20 min, and electrophoresis was carried out by adjusting the voltage to 25 V and the current to 300 mA (~0.7 V/cm) for 15 min. After electrophoresis, the slides were washed gently to remove any alkali and detergents that would interfere with ethidium bromide staining, using neutralisation buffer (0.4 M Tris-HCl, pH 7.4) three times for 5 min. After neutralization, the slides were stained with 50 *μ*L of 3.3 *μ*g/mL ethidium bromide in distilled water and covered with a coverslip. The slides were placed in a humidified air-tight container, to keep the gel from drying, until analysis. Three slides were prepared per assay, and 50 nuclei were counted per slide.

Slides were examined using an epifluorescence microscope, Zeiss Axioskop 20 (Carl Zeiss, Microscope Division, Oberkochen, Germany), equipped with a short arc mercury lamp HBO (50 W, 516–560 nm, Zeiss), using a 20X dry objective. Fifty randomly selected comets on each triplicate slide were scored with a Pulmix TM 765 camera (Kinetic Imaging, Liverpool, UK) connected to a Komet 3.0 image analysis system (Kinetic Imaging). This software defined different parameters for image processing. Among these parameters, we chose the percentage of DNA in the tail for the evaluation of DNA damage. The percentage of DNA in the tail is linearly related to DNA break frequency [29].

#### 2.6.2. Confocal Microscopy

Apoptotic cell death was assessed by evidencing nuclear morphology alterations. At the end of various cell treatments, growth medium was supplied with 1.6 *μ*M Hoechst 33258 (final concentration) for 15 min at 37°C. Coverslips were then rinsed twice with PBS, fixed for 15 min with 70% ethanol, and stored at 4°C in PBS until analysis. Cells were imaged using a confocal laser-scanning microscope Leica TCS SP2 AOBS (Leica, Heidelberg, Germany). For fluorescence excitation, an UV laser at 351–364 nm was used. Optical sections were recorded using a 63x oil immersion objective. Images were collected in the 512 × 512 pixel format and processed by Leica confocal software.

### 2.7. Statistics

Three independent experimental series were conducted for each exposure condition, unless otherwise indicated. Controls corresponded to astrocytes not exposed to ethanol and grown in culture under the same conditions as those in the experimental series.

The results were expressed as mean ± SEM. The Mann-Whitney *U* test was used for statistical analysis. Findings with *P* < 0.05 were considered significant. The Kolgomorov-Smirnov test was employed to compare the distribution of the percentages of DNA in the tail. Values of *P* < 0.05 were considered to be significant.

## 3. Results

In the literature, several authors dissolved resveratrol in DMSO and did not observe any cytotoxicity with this solvent [15, 20, 21, 30]. Unfortunately, with our experimental conditions, that is, long-term exposure and the fragility of cerebral cells in primary culture, a moderate decrease in cell viability (15%) was observed when astrocytes were exposed to this vehicle. Finally, we tested an H_2_O/DMSO (50/50, V/V) mixture to solubilize resveratrol, as described by Olas et al. [31]. In these conditions, the ratio DMSO/culture medium was 0.0005%, and no toxicity was detected for astrocytes in primary culture (data not shown).

### 3.1. Resveratrol Photosensitivity

**Figure 1:**
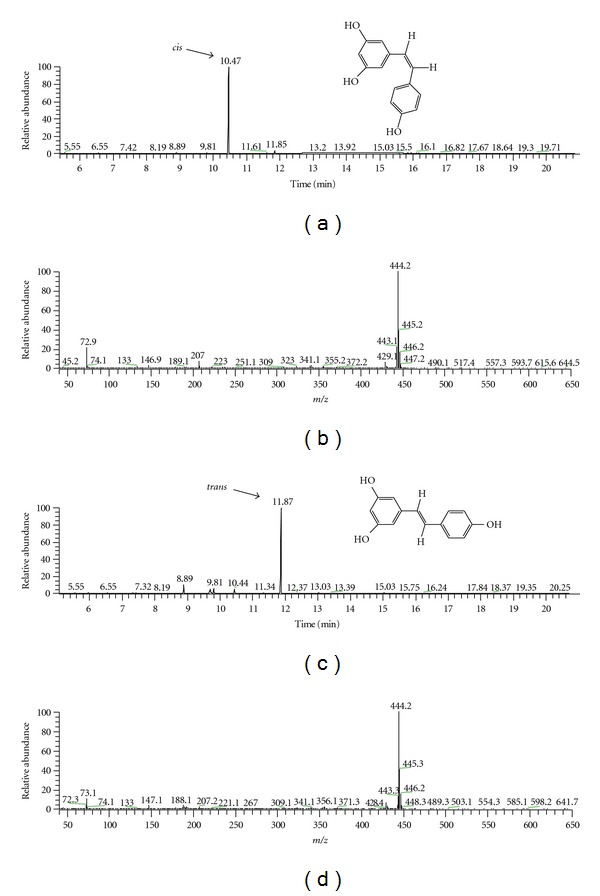
GC/MS analysis of resveratrol solubilized in an H_2_O/DMSO mixture. (a, c) show chromatograms obtained with samples placed either in light (a) or in darkness (c). (b, d) represent mass spectrum of *cis- *and *trans*-resveratrol, respectively. The analysis conditions are detailed in Section 2.

Resveratrol was only commercially available as the *trans*-isomer, the pharmacologically active form of this polyphenol. Resveratrol is a photosensitive compound, but only little information was available in the literature concerning precautionary measures against light exposure in order to avoid its* cis*-*trans* photoconversion and a possible inactivation of its protective action [10, 32]. It has been demonstrated that *trans*-resveratrol is susceptible to UV-induced isomerization in the *cis*-form, but only a few authors noted that they worked in darkness [10, 33, 34]. Is the *cis*-form active in protection against ethanol-induced damage? To answer this question, we induced a *trans-cis* isomerization of resveratrol by exposing an aliquot to laboratory light. The sample was then analyzed using GC-MS and the chromatogram obtained was compared to the one recorded with an identical aliquot placed in the dark. With our GC/MS analysis conditions, the peak observed for the aliquot placed in the dark has a retention time of 11.87 min (Figure 1(c)) whereas, with the light-exposed sample, the peak corresponding to the *cis*-isomer has a retention time of 10.47 min (Figure 1(a)).

**Figure 2:**
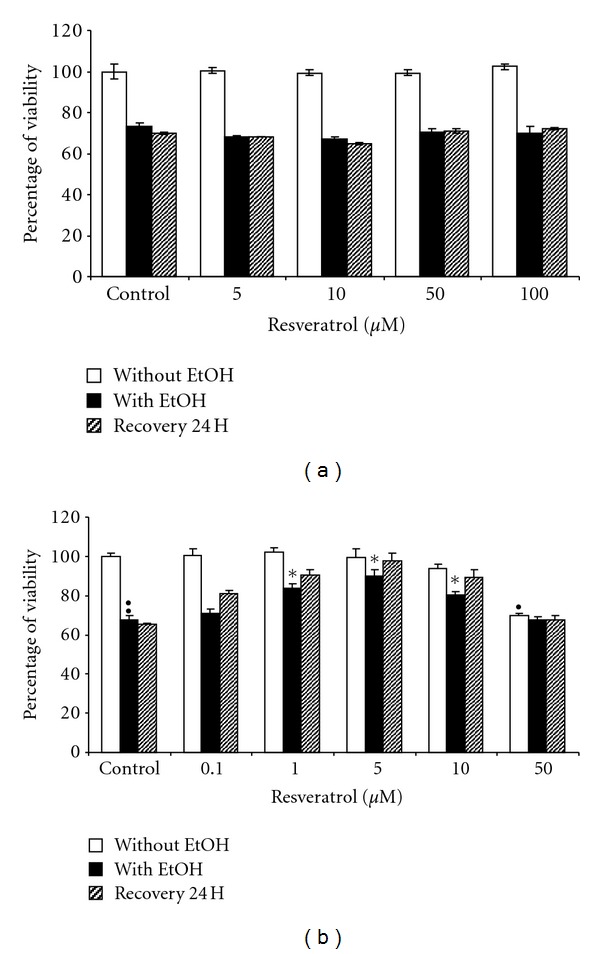
Astrocyte variation in viability after 1 h of pretreatment with various concentrations of *cis*-resveratrol (a) or *trans*-resveratrol (b) followed by a 3-day exposure to 20 mM ethanol in presence of resveratrol. Determinations were made directly after the exposure or after 24 h of recovery in fresh culture medium. Cytotoxicity was determined by MTT assay, and control without resveratrol and ethanol represents 100% viability. Data shown were obtained in a typical experiment representative of three independent experiments and are expressed as mean ± SEM, *n* = 5 (^•^ *P* < 0.01 resveratrol-treated cells versus control, ^••^ *P* < 0.001 ethanol exposed cells versus control, *P < 0.05, Res + ethanol treated cells versus control ethanol, Mann-Whitney *U* test). Cell exposure conditions are detailed in Section 2.

To assess the neuroprotection offered by these two isomers, we tested the effects of a pretreatment of astrocytes with *cis*- or *trans*-resveratrol on the toxicity induced by a long-term exposure to ethanol (20 mM, 3 days). No effect was observed when *cis*-resveratrol was added to cell culture, whatever the concentrations tested (Figure 2(a)), whereas the *trans*-isoform induced a slight decrease in ethanol toxicity (Figure 2(b)). 

### 3.2. Effects of *Trans*-Resveratrol on Cytotoxicity Induced by a Long-Term Exposure to Ethanol

To determine which doses were most efficient against cellular damage induced by a long-term exposure to ethanol, a wide range of *trans-*resveratrol concentrations (from 0.1 to 100 *μ*M) were tested. Astrocytes were pre- (1 h) and coincubated with these various concentrations of *trans-*resveratrol in the presence or absence of 20 mM ethanol for 3 days. After exposure, astrocytes were replaced in fresh medium for 24 h to evaluate cell recovery. As previously described and shown in Figure 2(b), viability of cultured astrocytes exposed to ethanol was significantly reduced by approximately 30% when compared to control without ethanol (*P* < 0.001, Mann-Whitney *U* test). Moreover, no change in viability was observed after the recovery period (Figure 2(b)). 

Treatment of astrocytes with low concentrations of *trans*-resveratrol (0.1–5 *μ*M) did not induce any change in cell viability when compared to control without *trans-*resveratrol (Figure 2(b)). While a slight decrease in cell viability was observed with *trans*-resveratrol 10 *μ*M, a loss of viability of 25% was observed with a 50 *μ*M treatment (Figure 2(b), *P* < 0.01 Mann-Whitney *U* test) which reached to 70% when astrocytes were treated with *trans-*resveratrol 100 *μ*M (data not shown). 

No significant toxicity was observed for the lowest concentration of *trans-*resveratrol tested (0.1 *μ*M), but unfortunately no protection towards ethanol damage was offered at this concentration (Figure 2(b)). Moreover, significant protection was provided with 1- to 10-*μ*M concentration of *trans-*resveratrol (Res1 or 5 or 10 + ethanol versus ethanol, *P* < 0.05, Mann-Whitney *U* test, Figure 2(b)). Indeed, in presence of ethanol the best protective effect was observed with a 5-*μ*M concentration of *trans-*resveratrol, and this concentration provided the best cell recovery (Figure 2(b)). In conclusion, a dose-dependent protection against ethanol-induced neurotoxicity was offered by *trans-*resveratrol at concentrations from 0.1 to 5 *μ*M. In the same manner, this polyphenol provided dose-dependent cell recovery after 24 h in fresh medium.

For concentrations of *trans-*resveratrol higher than 5 *μ*M, protective effects against ethanol-induced toxicity were decreased in a dose-dependent manner. For example, in presence of ethanol, 50 *μ*M *trans-*resveratrol induced a substantial decrease in the viability percentage, which was nearly identical to those observed for ethanol alone, whether immediately after the stress or after a 24-h recovery period (Figure 2(b)).

### 3.3. Effects of *Trans*-Resveratrol on Genotoxicity Induced by a Long-Term Exposure to Ethanol

**Figure 3:**
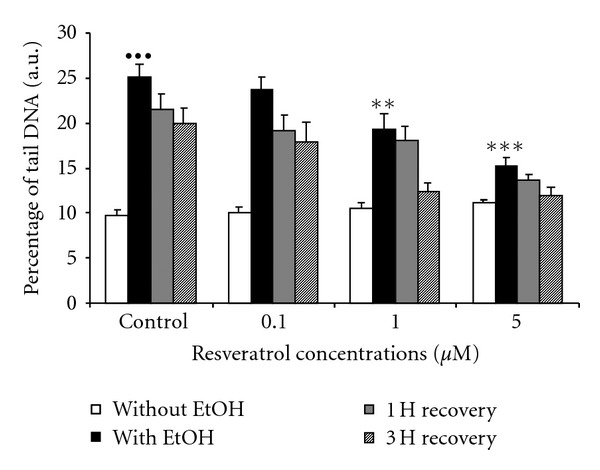
Influence of a 1 h pretreatment with various concentrations of *trans*-resveratrol on the level of DNA damage observed in astrocytes exposed to a 3-day treatment with 20 mM ethanol, followed or not by a 1 h or 3 h recovery period in fresh culture medium. DNA damage was evaluated using the comet assay and is expressed as percentage of DNA in the tail. The figure shows the mean results of three independent experiments. Fifty cells were randomly examined in triplicate for each condition, and results are expressed as mean ± SEM (^•••^ *P* < 0.0001 ethanol exposed cells versus control, **P < 0.05, ***P < 0.001 Res + ethanol-treated cells versus control ethanol, Mann-Whitney *U* test). Experimental conditions are detailed in Section 2. a.u.: arbitrary units.

Considering results previously obtained with the MTT assay, the protective effects offered by *trans-*resveratrol against DNA damage were only studied for concentrations from 0.1 to 5 *μ*M. According to the results of the comet assay, the percentage of DNA in the tail was significantly increased after a 3-day exposure to ethanol when compared to control cells without ethanol. The percentage of tail DNA in the cells exposed to ethanol was more than twice as high as the control values (25.2 versus 9.7, *P* < 0.0001, Mann-Whitney *U* test, Figure 3). Nevertheless, contrary to results observed with the MTT test, the comet assay showed a slight decrease in the DNA strand breaks during the recovery periods, when compared to the exposed conditions with no recovery, particularly for the 3 h recovery time where values were significantly different (25.2 for cells exposed to ethanol versus 20.0 for cells with a 3 h recovery period, *P* < 0.05, Mann-Whitney *U* test, Figure 3). 

No genotoxicity was observed when *trans*-resveratrol was added to culture medium at concentrations ranging from 0.1 to 5 *μ*M (Figure 3). In addition, when cells were exposed to ethanol in presence of *trans-*resveratrol, a significant and dose-dependent decrease in DNA damage was observed for the 1-*μ*M and 5-*μ*M concentrations (25.2 for cells exposed to ethanol versus 19.4 for cells exposed to Res1 + ethanol, *P* < 0.05, and 25.2 for cells exposed to ethanol versus 15.1 for cells exposed to Res5 + ethanol, *P* < 0.001, Mann-Whithney *U* test, Figure 3). Moreover, replacing cells in fresh medium after the stress enhanced cell recovery when *trans-*resveratrol was present in the intoxication medium. After a 3 h recovery period, nearly complete recovery was observed for cells pretreated with 1 *μ*M and 5 *μ*M *trans-*resveratrol when compared to control (Figure 3).

**Figure 4:**
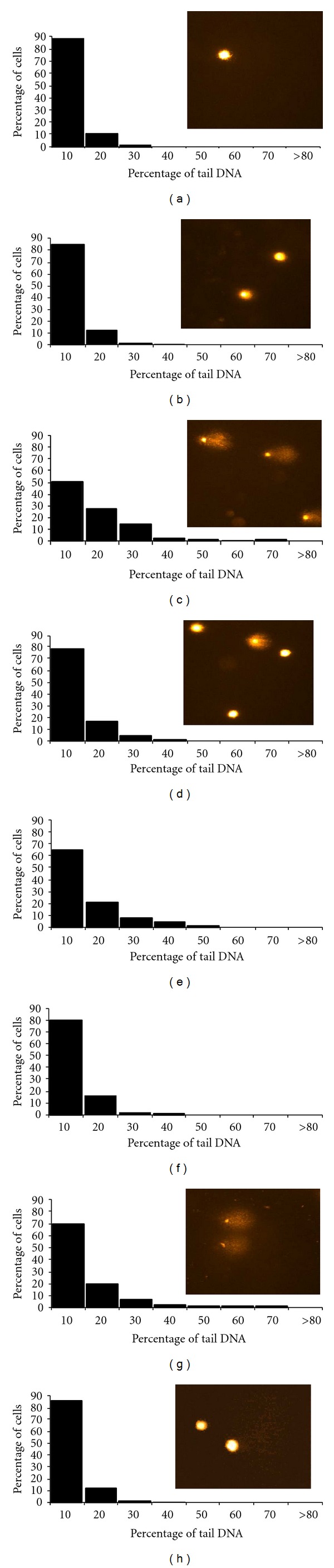
Histograms of the distribution of comet tail DNA obtained after a 3 day exposure of astrocytes to 20 mM ethanol, preceded or not by a 1 h pretreatment with resveratrol (5 *μ*M) and followed or not by a 1 h or 3 h recovery period in fresh culture medium. (a) Control; (b) pretreatment with resveratrol; (c) 3-day exposure to ethanol; (d) pretreatment with resveratrol followed by exposure to ethanol; 1 h recovery (e) and 3 h recovery (g) of astrocytes after ethanol exposure; 1-h recovery (f) and 3 h recovery (h) of astrocytes after ethanol exposure when cells were first pretreated with resveratrol. (a) versus (c), (d) versus (c), (f) versus (e), *P* < 0.0001, Kolmogorov-Smirnov test. Experimental conditions are detailed in Section 2. Insets show representative micrographs of “comet,” corresponding to the various cell treatments described in the legend.

Figure 4 showed interesting changes in the distribution of tail DNA: in control conditions, more than 85% of cells had tail DNA between 0% and 10% (Figure 4(a)), whereas significant modifications in the distribution of tail DNA were observed after a 3-day exposure to ethanol (Figure 4(c)), with a progressive distribution towards higher percentages of tail DNA (control versus ethanol, *P* < 0.0001, Kolgomorov-Smirnov test). When cells were replaced in fresh medium without ethanol for a 1 or 3 h recovery period, the previously described decrease in DNA strand breaks was confirmed. This recovery process was time dependant but incomplete since only 69.1% of cells had tail DNA between 0% and 10% after 3 h of recovery (Figure 4(g)) compared to 88.2% of cells in control conditions (ethanol + 3 h recovery versus control, *P* < 0.0001, Kolgomorov-Smirnov test, Figure 4(a)). When cells were pretreated with 5 *μ*M *trans-*resveratrol, no toxicity was observed for these astrocytes (Figure 4(b)) when compared to control (Figure 4(a)). Moreover, there was less DNA damage after 3 day exposure to ethanol and in presence of *trans-*resveratrol (Figure 4(d)) since 78.2% of cells had tail DNA between 0% and 10% compared to 50.4% for cells exposed to ethanol alone (Res5 + ethanol versus ethanol, *P* < 0.0001, Kolgomorov-Smirnov test, Figure 4(c)). In addition, the recovery process was also improved; after 1h of recovery, 80% of cells had tail DNA between 0% and 10% (Figure 4(f)), whereas without *trans-*resveratrol only 65% of cells had tail DNA between 0% and 10% (Figure 4(e)), (Res5 + ethanol + 1 h recovery versus ethanol + 1 h recovery, *P* < 0.0001, Kolgomorov-Smirnov test). After 3 h of recovery, it could be considered that the recovery was complete when cells were pretreated with 5 *μ*M *trans-*resveratrol (88.2% and 85.3% of cells with tail DNA between 0% and 10%, for control (Figure 4(a)) and Res5 + ethanol + 3 h recovery (Figure 4(h)), respectively. Indeed the histograms of distribution are not only not statistically different, but also nearly identical for these two conditions. 

**Figure 5:**
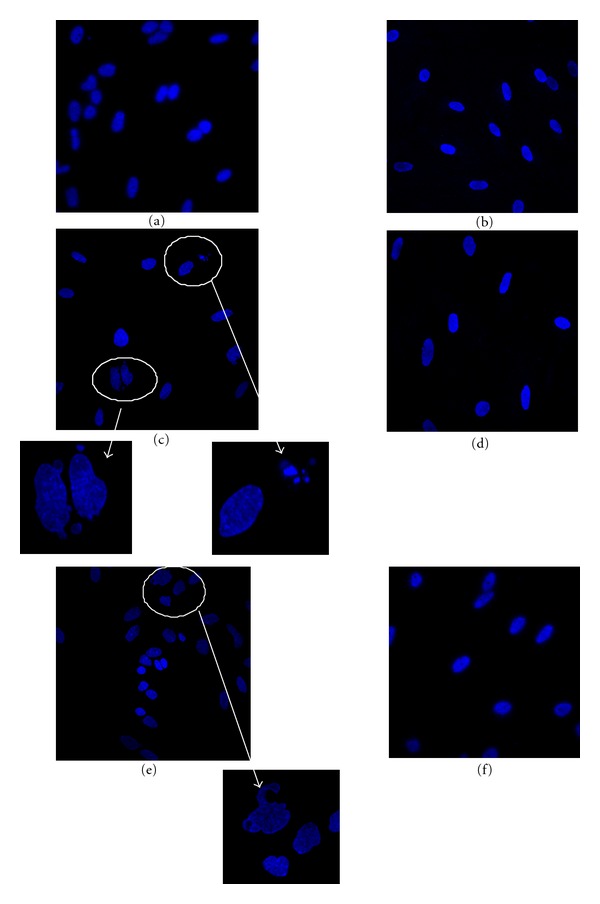
Effect of treatment with 5 *μ*M resveratrol on ethanol-induced nuclear damage in primary cultures of astrocytes. Nuclei were labeled with Hoechst 33342 as described in Section 2. Figure 5 shows representative fluorescent microscope images evidencing the nuclear morphology of astrocytes in control conditions (a), exposed to 5 *μ*M resveratrol for 3 days (b), exposed to 20 mM ethanol for 3 days without (c) or with a recovery period (e), or exposed to 20 mM ethanol for 3 days, with pre- and cotreatment with 5 *μ*M resveratrol (d) and followed by a recovery period (f). In zoom sections of micrographs, arrows point nuclear blebs and nuclear fragmentation. All images are representative fields of at least three independent experiments carried out in duplicate.

Nuclear damage was also evidenced based on nuclear morphology. Astrocyte staining with nucleic acid dye Hoechst 33342 was performed after various treatments. Cells were then analyzed using laser confocal microscopy. In contrast to regular, blue nuclei observed in viable astrocytes of the control group (Figure 5(a)), nuclei with membrane blebbings, irregular shapes, and apoptotic bodies were evidenced in cells exposed to 20 mM ethanol for 3 days (Figure 5(c)). These deleterious effects lasted after the recovery period since blebs of the nuclear membranes were always detectable (Figure 5(e)). Astrocytes pretreatment with 5 *μ*M *trans-*resveratrol did not induce any toxicity (Figure 5(b)). Moreover, the treatment with this *trans-*resveratrol concentration decreased damaged nuclei induced by ethanol, particularly after the recovery period where no abnormal nuclear morphology was evidenced (Figures 5(d) and 5(f), resp.).

## 4. Discussion

Normal cell metabolism results in a continuous generation of reactive oxygen species (ROS) that is strictly controlled by antioxidant mechanisms. However, in some circumstances, oxidative stress can occur as a result of increased exposure to normal metabolites of oxidative metabolism. This can happen when ROS production is stimulated by the metabolism of certain toxicants and/or when the production or the bioavailability of antioxidants is affected by such agents. Consequently, the physiological balance between oxidants and antioxidants can be directly or indirectly modified by a toxicant. Among these toxicants, ethanol is known to produce free radicals during its metabolism [4, 9, 35]. Particularly, chronic exposure to ethanol is known to induce cellular and nuclear damage in cerebral cells, mediated by a free radical pathway [5, 6, 36, 37]. 

The role of polyphenol obtained from diet in protection against oxidative stress is a topic of continuing interest and some controversy. 

Several *in vivo* and in *vitro* studies have reported measurable concentrations of *trans*-resveratrol after administration to animals or exposure to cells. For instance, Bertelli et al. [38] showed that single or prolonged administration to rats of red wine with a known *trans-*resveratrol content led to its accumulation in blood and various organs. In addition, Vitrac et al. [39] demonstrated that ^14^C-labeled *trans*-resveratrol is absorbed, metabolized and distributed in the whole body of mice orally treated with this polyphenol. Moreover, because of its high lipid solubility, resveratrol might be deposited in tissues with high lipid content such as brain and the nervous system [40], making this polyphenol a first-class compound for neuroprotective studies. Resveratrol transport from plasma to intracellular targets seems to involve both passive diffusion and a carrier-mediated process [41, 42]. 

Concerning cerebral cells, Guo et al. [43] described useful protection offered *in vivo* by a large range of resveratrol concentrations against genotoxicity induced by acute and chronic ethanol exposure. On the contrary, several authors pointed the importance of resveratrol concentrations towards cellular protection. Thus, they described a neuroprotective effect for low concentrations of resveratrol when high doses of this polyphenol induced cell toxicity [44–46]. Recently, Quincozes-Santos et al. [47] evidenced that the nature of the stress could be more important than the resveratrol concentration. They demonstrated that under intense but short oxidative conditions resveratrol was able to protect C6 glioma cells against H_2_O_2_ insult while under less intense but lasting oxidative insult, resveratrol had an opposite effect, potentiating the H_2_O_2_-induced damage and resulting in a prooxidant effect.

In this study, brain protection offered by *trans-*resveratrol, against ethanol-induced toxicity, was investigated. In order to avoid its photoisomerization leading to an inactive isomer (Figure 2(a)), *trans*-resveratrol has to be carefully kept in the dark throughout the experiments, from sample preparation to cell treatment. 

Contradictory information concerning the effects of resveratrol treatment has been reported in the literature. Large variations in experimental systems such as cellular models, resveratrol concentrations, incubation durations, and so forth could explain these differences. Therefore, it seems important to test a large range of resveratrol concentrations to define the optimal concentration offering cellular protection with our own experimental system. The concentrations used for evidencing the protective effects of resveratrol against cellular damage induced by ethanol have been the subject of preliminary investigations. In our experimental conditions, treatment of astrocytes for 3 days with low concentrations of resveratrol (from 0.1 to 10 *μ*M) did not induce any change in viability, whereas a toxic effect was observed when the highest concentrations of resveratrol (50 and 100 *μ*M) were added to cells, characterized by a dose-dependent cell mortality rate higher than 30%. These results are in accordance with those reported by Quincozes-Santos et al. [47].

As previously described [5], a chronic exposure of astrocytes to ethanol led to a significant loss of viability, as evidenced by the MTT assay. Moreover, when cells were replaced in fresh medium for a 24 h poststress period, no recovery was observed. These results revealed durable alterations in the normal functioning of astrocytes, particularly for the respiratory and energetic process measured by the MTT assay. The protection offered by *trans-*resveratrol against ethanol cytotoxicity was dose-dependent and only observed for the lowest concentrations, but the most valuable result concerned the effect of this polyphenol on the recovery of astrocytes; cell recovery was dose dependent and complete for 5 *μ*M resveratrol.

The potential genotoxicity of long-term ethanol administration to astrocytes was investigated using comet assay. This method provides fast results and requires only a few cells, so it seems suitable for analysis in primary culture, which requires continual new preparations.

The percentage of tail DNA was increased 2.5-fold after long-term exposure to ethanol. Moreover, after a 1 or 3 h recovery period in fresh medium, these DNA strand breaks were only partially self-repaired. These results demonstrated the genotoxicity of ethanol. In addition, it is generally considered that DNA damage caused by chemicals increased the risk of mutation and cancer, even though the DNA damage may be self-repaired [48]. 

When astrocytes were exposed to ethanol for 3 days, *trans-*resveratrol treatment induced both a decrease in DNA damage and an enhancement of cell recovery in a dose-dependent manner. The effective concentration of *trans-*resveratrol that both reduced DNA strand break formation and enhanced DNA repair was 5 *μ*M.

Consistent with these results, confocal laser microscopy images of astrocytes stained with Hoechst 33342 allowed visualization of apoptotic nuclei when cells are exposed for 3 days to 20 mM ethanol. Interestingly, treatment with 5 *μ*M resveratrol completely prevented nuclear morphology alterations induced by ethanol treatment since neither membrane blebbings nor apoptotic bodies were evidenced in these conditions.

Several mechanisms may underlie *trans-*resveratrol-induced protection of astrocytes against ethanol neurotoxicity. *Trans-*resveratrol has been shown to possess helpful free-radical scavenging properties [19, 20, 49–51], and ethanol exposure is known to induce the generation of reactive free radicals *in vitro* [8, 9, 52] as well as *in vivo* [53, 54]. Therefore, it is reasonable to assume that *trans-*resveratrol might have protective effects on ethanol-induced oxidative DNA damage, by quenching free radicals generated during its brain metabolism.

In conclusion, we clearly evidenced that *trans*-resveratrol could markedly decrease cell mortality and levels of DNA strand breaks induced by long-term ethanol exposure of astrocytes in primary culture. Moreover, we demonstrated that this polyphenol promoted poststress cell recovery. Nevertheless, this expected significant protection should be weighted against the restrictive conditions of resveratrol treatment since elevated levels and/or long-term exposure with this compound could contribute to enhance cerebral damage. 

Although the relevance of our findings to *in vivo* clinical situations remains to be demonstrated, our results suggest that caution is necessary with therapeutic use of *trans-*resveratrol since high level of this compound could lead to the appearance of adverse effects on the brain.
